# The Surgical Management of Thyroid Cancer

**DOI:** 10.5041/RMMJ.10142

**Published:** 2014-04-28

**Authors:** Sara A. Morrison, Hyunsuk Suh, Richard A. Hodin

**Affiliations:** Department of Surgery, Massachusetts General Hospital, Boston, MA, USA

**Keywords:** Controversies in thyroid surgery, papillary microcarcinoma, thyroid cancer

## Abstract

Thyroid cancer has been increasing in incidence, with the number of reported cases in the US rising by 25% over the last 3 years. With growing technological advances in the field and improved contributions of diagnostics, surgical decision-making and operative planning have taken on new challenges. Herein, we review the current clinical practice recommendations and active areas of surgical controversy, reflective of the most recently published professional consensus guidelines and a systematic review of the literature.

## BACKGROUND

There are approximately 63,000 reported cases of thyroid carcinoma annually in the United States, representing roughly 4% of all documented malignancies.^1^ Diagnosis typically stems from work-up of a thyroid nodule. Data from the Framingham study suggests that palpable thyroid nodules are present in 4% of the US population,^2^ but non-palpable nodules may exist in up to 67% of the population. Such nodules are often found incidentally secondary to the rising use of imaging modalities in medical settings. The large majority of thyroid nodules are benign, with an overall reported risk of malignancy from 5% to 15%.^3^ Surgery remains the mainstay therapy for most patients with thyroid cancer. With increasing incidence of thyroid nodules, improved diagnostic imaging modalities, molecular markers, tumor biology, emerging technologies in thyroid surgery (e.g. minimally invasive, video-assisted thyroidectomy, endoscopic or robotic thyroidectomy) and postoperative adjuvant therapies, the field of thyroid cancer and the importance of surgeon’s knowledge and experience in the overall management of thyroid cancer have become very dynamic and challenging.

## EVALUATION AND DIAGNOSIS

Evaluation is typically pursued for patients with thyroid nodules larger than 1 cm, as well as in patients who may have smaller nodules but carry a family history of thyroid cancer, a personal history of head or neck radiation, or who present with concerning features on imaging. Additionally, all nodules which are found incidentally to be positive during PET imaging should be further evaluated as these nodules are reported to carry a 30% risk of malignancy.^4^

Many professional organizations (e.g. National Comprehensive Cancer Network (NCCN); American Thyroid Association (ATA); American Association of Clinical Endocrinologists (AACE)) have published guidelines for the evaluation of thyroid nodules. These guidelines can be summarized to include the following: sound clinical assessment, ultrasound evaluation, TSH level, and biopsy/cytologic evaluation (if indicated based on the size and imaging characteristics) to assess for malignancy. Ultrasound evaluation and fine-needle aspiration (FNA) biopsy are the gold standard for thyroid cancer diagnosis.

A thorough history and physical exam remains a critical step in thyroid nodule evaluation. Symptoms of thyrotoxicosis or hypothyroidism, local compressive symptoms, voice changes, and/or the presence of dysphagia should be elicited. In addition, patients should be questioned regarding a personal history of head or neck irradiation, or a family history of either thyroid cancer or other familial syndromes, as there are several known to be associated with an increased risk for thyroid cancer (Table 1).^5^,^6^

**Table 1.** Familial Syndromes Associated With Thyroid Cancer.^5^,^6^

**Familial Syndrome**	**Clinical Characteristics**	**Inheritance Pattern**	**Involved Gene**	**Thyroid Cancer**
Multiple endocrine neoplasia (MEN) 2A and 2B	2A: MTC, pheochromocytoma, primary hyperparathyroidism 2B: MTC, pheochromocytoma, neurofibromatosis	Autosomal dominant	RET proto-oncogene	MTC
Familial medullary thyroid cancer	Isolated medullary thyroid cancer	Autosomal dominant	RET proto-oncogene	MTC
McCune Albright syndrome	Precocious puberty, polyostotic fibrous dysplasia, café-au-lait spots	Mosaic	GNAS1	FC
Familial adenomatous polyposis (FAP)	Intestinal adenomatous polyps	Autosomal dominant	APC tumor suppressor gene	PTC
Gardner syndrome	Variant of FAP, with addition of desmoid tumors, ostomas	Autosomal dominant	APC tumor suppressor gene	PTC
Carney complex	Hyperpigmentation of mucosa, schwannomas, pituitary and testicular tumors	Autosomal dominant	PRKARI	PTC
Cowden syndrome	Hamartomas of multiple organs	Autosomal dominant	PTEN tumor suppressor gene	FC
Werner syndrome	DM, cataracts, muscle atrophy, premature aging	Autosomal dominant	WRN	FC, PTC

* DM, diabetes mellitus; FC, follicular thyroid cancer; MTC, medullary thyroid cancer; PTC, papillary thyroid cancer

On exam, it is important to note both the character and size of the nodule in question, whether the nodule is fixed or mobile, as well as to assess for the presence of neck tenderness, tracheal deviation, or potentially concerning cervical lymphadenopathy. All patients with dysphonia or prior history of cervical or thoracic operations with concern for recurrent laryngeal nerve injury should be evaluated with direct laryngoscopy to assess vocal cord function and for preoperative planning.

An ultrasound (US) of the neck should be obtained in order to evaluate the nodule in question and the remainder of the gland for synchronous findings. Concerning features on US include nodules that are hypoechoic, have increased vascularity, contain calcifications, or have irregular shape (classically taller than wide) and borders, or evidence of extra-thyroidal extension or invasion of surrounding structures. The remainder of the neck (bilateral central and lateral compartments) should be assessed to evaluate for the presence of abnormal lymph nodes.^2^

Modeled after the Breast Imaging Reporting and Data System (BIRADS), developed by the American College of Radiology, the Thyroid Imaging Reporting and Data System (TIRADS) was designed in order better to standardize the classification of thyroid lesions and allow for enhanced communication among treating physicians in co-ordinating clinical management. The TIRADS scale ranges from 1 to 6, a score of 1 representing a normal thyroid gland, a 2 signifying a benign condition with no risk of malignancy, 3 being used for nodules that are likely benign, with a corresponding risk of malignancy of less than 5%, 4 denoting a suspicious nodule, with malignancy risk ranging from 5% to 80%, 5 being used to describe a nodule that is likely malignant, with a greater than 80% estimated likelihood, and, similarly to the BIRADS scale, a 6 signifying known malignancy, proven by prior biopsy. In a prospective study of nearly 2,000 lesions, the TIRADS scale was found to have a sensitivity and specificity of 88% and 49%, respectively. In addition, they found a positive predicative value of 49%, negative predictive value of 88%, and accuracy of 94%. As a result of these and similar study findings, the TIRADS method of data reporting is gaining in use within clinical practice.^7^

All patients should be assessed with a baseline TSH level. A low serum TSH should be followed by a radio-iodine scan to determine the functional status of the nodule. Hyperfunctioning nodules are rarely malignant and can be monitored. Iso- or hypo-functioning nodules should undergo further investigation with a FNA biopsy based on the size, appearance, and clinical suspicion (Table 2).^3^

**Table 2.** American Thyroid Association Recommendations for Fine-Needle Aspiration (FNA) Biopsy.^3^

**Clinical or Sonographic Nodule Features**	**Size at which FNA is Recommended**
Increased risk medical history: family history of thyroid cancer, personal history of prior thyroid cancer, head or neck radiation	>5 mm
Sonographic or clinically suspicious cervical lymphadenopathy	All
Presence of microcalcifications	≥1.0 cm
Purely cystic nodule	FNA not recommended
Spongiform nodule	≥2.0 cm or continued monitoring
Mixed cystic and solid nodule	≥1.5 cm if suspicious sonographic features present, otherwise ≥2.0 cm
Solid nodule	≥1.0 cm

Prior to the use of routine FNA biopsies in the work-up of thyroid nodules, the incidence of malignancy found following surgery was as low as 14%. The use of FNA in current clinical practice has resulted in post-surgical pathology findings of malignancy in over 50% of specimens.^7^ The Bethesda System for Reporting Thyroid Cytopathology (TBSRTC) was developed in order to allow pathologists among varying institutions to communicate results to clinical care-takers with widely understood descriptors. Results of FNA biopsies are broken down into the following categories with the corresponding risks of malignancy: non-diagnostic or unsatisfactory (1%–4%), benign (0%–3%), atypia of undetermined significance or follicular lesion of undetermined significance (AUS/FLUS; 5%–15%), follicular neoplasm or suspicious for a follicular neoplasm (FN/sFN; 15%–30%), suspicious for malignancy (60%–75%), and malignant (97%–99%).^8^

If the biopsy specimen is non-diagnostic, the biopsy should be repeated with US guidance. Biopsies that are persistently non-diagnostic should undergo surgical removal of the involved lobe as there is an 8% risk of malignancy. Nodules with benign biopsy results can be followed yearly, as the false negative rate for such lesions is approximately 2%. Biopsies should be repeated for nodules which demonstrate interval growth.^9^,^10^

Conversely, malignant findings on biopsy should prompt referral for total thyroidectomy. If the available pathology is suspicious for malignancy, these patients may undergo lobectomy followed by a completion thyroidectomy, as indicated, versus a total thyroidectomy, depending upon clinical suspicion. Biopsies reported as “atypical or follicular lesion of undetermined significance” should be repeated in 3–6 months, and, if this diagnosis remains on the repeat specimen, ipsilateral thyroid lobectomy should be pursed, as these lesions carry a 19% risk of malignancy.^9^,^10^ About 20% of FNA biopsies will be indeterminate as defined by the Bethesda criteria III (AUS/FLUS) and IV (FN/sFN) leading to unnecessary diagnostic surgeries for most patients as only 5%–30% prove to be malignant on final pathology.^11^

In order to improve and complement FNA diagnosis accuracy, many diagnostic modalities have been investigated. Among them, molecular markers have shown some promise, and there are several commercially available genetic markers that are being utilized and integrated into the practice guidelines. Currently there are two commonly used forms of molecular testing: genetic mutational panel and gene expression profiling.

### Mutational Panels

AsuragenmiR Inform (Austin, TX, USA) mutation analysis assay and Thyroid Cancer Mutation Panel by Quest Diagnostics (Madison, NJ, USA) are the two main commercially available mutational tests which test for known genetic alterations such as BRAF, RAS, RET/PTC, and PAX8/PPARγ. These mutational panels are highly specific for malignancy; however, due to the low overall frequency of these mutations in thyroid cancers, negative results do not rule out cancer. Therefore, mutational panel tests are considered a “rule-in” test. If a preoperative mutational test is positive, the nodule should be considered malignant, and total thyroidectomy should be recommended.^12^,^13^

### Gene Expression Profiling

The most widely known gene expression profiling test is Afirma Gene Expression Classifier (Veracyte, San Francisco, CA, USA), and, with its recent clinical validation by Alexander et al., Afirma is already being utilized in many clinical settings. The Afirma Gene Expression Classifier (GEC) is an RNA-based assay that utilizes FNA samples to evaluate 167 molecular genes associated with benign nodules based on their proprietary algorithm. Unlike the mutational panel testing, Afirma testing is considered a “rule-out” test since the test has a high negative predictive value in distinguishing benign nodules. However, a positive result reported as “suspicious” carries only 38% risk of malignancy.^14^

In all, these molecular tests should be utilized judiciously and should be considered as a complementary diagnostic tool in the management of thyroid nodules. In the future, molecular testing could become more cost-effective and accurate as a diagnostic tool while providing prognostic and therapeutic information.

## SURGICAL MANAGEMENT

### Papillary Thyroid Cancer

Total thyroidectomy is the gold standard for patients with a preoperative diagnosis of papillary thyroid cancer when the nodule is greater than 1 cm in size.^15^ Completion thyroidectomy is indicated in patients who have undergone prior lobectomy and are found on final pathology to have papillary thyroid cancer that is larger than 1 cm. The completion thyroidectomy should generally be performed within 6 months of the original procedure in order to minimize the risk of lymph node metastasis. On the other hand, a number of centers have demonstrated that low-risk patients with uninodular, large cancers that are confined to the thyroid gland can be treated with thyroid lobectomy or subtotal thyroidectomy with no compromise in oncological outcome. In cases of extra-thyroidal extension, all gross disease should be resected en bloc at the time of the initial operation. In the setting of suspected recurrent laryngeal nerve involvement, it is important to document the vocal cord function preoperatively with a direct laryngoscopy. At operation, the nerve should be dissected from the cancer whenever possible to preserve its function. However, if the nerve is completely encased or infiltrated, the nerve should be sacrificed regardless of its function. Of note, it is critically important for the surgeon to ensure the function of the contralateral recurrent laryngeal nerve in order to minimize the potential need for a tracheostomy.^6^ Less frequently, thyroid cancers can also involve the trachea, esophagus, and/or larynx. The extent of the disease should determine the potential for a curative resection, and in some of these cases a multidisciplinary approach with an otolaryngologist and/or thoracic surgeon may be helpful. In such cases of locally advanced papillary thyroid cancer, adjuvant therapy with external beam radiation and in some cases chemotherapy may be indicated.

Involvement of cervical lymph nodes in papillary thyroid cancer is frequent, reported to occur in up to 50% of patients.^15^ The role of neck dissection at the time of total thyroidectomy is somewhat controversial, however, since most of the nodal involvement is microscopic and does not affect overall survival. It is generally agreed upon that a therapeutic neck dissection should be pursued in the setting of well-differentiated thyroid cancer patients with clinically positive lymph nodes, whether in the central or lateral neck compartments.^15^ There is little evidence to support routine central or lateral neck dissections in the absence of clinically positive nodes found on pre-op exam and/or imaging.

### Follicular Thyroid and Hurthle Cell Cancer

Patients are typically diagnosed with follicular thyroid and Hurthle cell cancer following a lobectomy, as these variants are generally not able to be discerned from their benign counterparts on routine FNA biopsy. Completion thyroidectomy is indicated for all patients with invasive follicular thyroid cancer. A subset of patients with minimal capsular invasion may be treated with lobectomy alone, as these variants tend to behave similarly to benign follicular adenomas.^16^ Completion thyroidectomy is performed in all patients with Hurthle cell carcinoma. Prophylactic neck dissection is not done for follicular thyroid cancer, as the rates of lymph node metastasis are typically less than 10%.^16^ Therapeutic dissections are performed in the setting of biopsy-proven metastasis to either the central or lateral neck. The rate of lymph node involvement in Hurthle cell cancer, however, is considerably higher, and therefore the ATA guidelines suggest that prophylactic central neck dissection be considered in these cases. There is no evidence currently in the literature that such practice extends a benefit in terms of disease-free survival^3^,^16^

### Medullary Thyroid Cancer

Medullary thyroid cancer (MTC) comprises 4% of all thyroid malignancies. The majority of cases are sporadic in nature; approximately 20%–25% represent familiar/hereditary syndromes.^17^ Diagnosis is commonly made by FNA biopsy with specific staining for the presence of calcitonin in the tissue specimen. All patients with a diagnosis of medullary thyroid cancer must be evaluated for multiple endocrine neoplasia (MEN) 2 and be ruled out for the synchronous presence of pheochromocytoma prior to scheduling thyroid surgery. This can be done via a negative screen for plasma free metanephrines, a 24-hour urine collection for metanephrines and normetanephrine, or a negative adrenal CT or MRI. Other preoperative work-up for medullary cancer patients include measurements of serum calcium, and calcitonin levels, as well as the carcinoembryonic antigen (CEA) level, a tumor marker commonly associated with a number of cancers, including endocrine, liver, and intestinal cases. RET proto-oncogene analysis should be offered to all patients with a history of either medullary thyroid cancer, MEN2, or primary C-cell hyperplasia.^18^

Total thyroidectomy is recommended for all patients with medullary thyroid cancer in order completely to remove the C-cells that are the source of this neoplasm. Occult disease in cervical lymph nodes is very common in patients with MTC and has been reported to be as high as 75%. Accordingly, prophylactic central neck dissections are routinely performed in MTC. Lateral neck dissection is only performed if there is clinical evidence of nodal involvement.^18^ Patients with locally advanced disease with distant metastasis may benefit from a debulking or palliative operation in order to prevent local neck symptoms. In addition, debulking surgery in MTC can lead to better control of the serum calcitonin levels, a hormone that can cause symptoms such as diarrhea. Patients with known genetic predisposition to MTC generally require a prophylactic total thyroidectomy based on the international guidelines.

### Anaplastic Thyroid Cancer

Anaplastic thyroid cancer (ATC) represents approximately 1% of all thyroid cancers. It is a rare but highly lethal cancer with a reported 1-year survival of less than 10%.^19^ Diagnosis is usually made by FNA biopsy, but in certain cases a core or open biopsy may be necessary, especially when trying to rule out lymphoma. At the time of presentation, less than 20% of patients with anaplastic thyroid cancer will have a tumor that remains confined to the thyroid gland. Surgical resection followed by adjuvant treatments in this select subset of patients has been shown to prolong survival.^20^

In the event that patients present with surgically resectable disease, without distant metastasis, treatment plans are multi-modal and include surgery, radiation, with or without the addition of chemotherapy. Aggressive surgery for ATC is especially worthwhile when the disease is unilateral in location. Current clinical trials have investigated the use of a combination of doxorubicin and cisplatin, in addition to docetaxel or paclitaxel in this setting. These agents have demonstrated a response in approximately 20% of patients.^19^,^21^

In cases of impending airway compromise, tracheostomy or tracheal stenting should be performed expediently. Surgery is controversial in patients with extra-thyroidal disease and has not been shown to extend survival, as it is frequently impossible fully to resect all gross disease. Rates of essential structure involvement are considerably high; tracheal invasion is present in 69% of patients, esophageal invasion in 55%, and carotid artery involvement in 39% of patients.^21^ Tracheostomy is not generally recommended in patients without imminent airway compromise. Neo-adjuvant therapy may be pursued in this setting in the hopes of downsizing the tumor, and potentially de-escalate the extent of surgery.

## TOTAL THYROIDECTOMY SURGICAL APPROACH

The surgical incision site is marked preoperatively along a natural skin crease, approximately 1–2 fingerbreadths above the sternal notch. Once in the operating room, the patient is placed in the supine position, with an inflatable bag or roll placed under the shoulders in order to extend the neck. The patient’s arms are tucked. A nerve monitor may be used at the discretion and preference of the operating surgeon.

A transverse skin incision is made and taken down through the subcutaneous tissue. The platysma is divided, and subplatysmal flaps are raised creating a plane that extends from the thyroid cartilage superiorly to the sterna notch inferiorly, and between the carotid arteries laterally. The cervical fascia is divided along the median raphe, and then the sternohyoid and sternothyroid muscles are retracted laterally. Except in cases of tumor invasion, these muscles do not require division. If there is tumor involvement of the overlying strap muscles, one of both should be divided and included in the specimen in order to accomplish an en bloc resection of the cancer. The thyroid gland is retracted anterio-medially during the dissection in order to assist in identification of key lateral structures. A superior-to-inferior approach is taken by most surgeons and represents a safe and efficient way to conduct the operation. The superior pole vessels are isolated and divided close to the capsule of the gland so as to minimize the risk of injury to the external branch of the superior laryngeal nerve. Vascular control can be accomplished through the use of suture ligation, clips, or a variety of energy devices.

Once the superior pole is taken down, one can identify the tubercle of Zuckerkandl and mobilize this part of the gland from its lateral and posterior position. This approach will almost always provide good exposure and access to the superior parathyroid gland, which should be maintained along with its blood supply. In addition, one can then readily identify the recurrent laryngeal nerve (RLN) which uniformly enters the trachea just inferior to the cricothyroid membrane. Branches of the inferior thyroid artery are divided close to the thyroid capsule so as to minimize the risk to the RLN and the blood supply to the neighboring parathyroid glands. The inferior pole of the gland is then mobilized, exposing the anterior surface of the trachea. With the thyroid lobe almost fully mobilized, the ligament of Berry is carefully separated from the recurrent laryngeal nerve and divided. One should then identify the pyramidal lobe and follow it to its most superior extent in order to resect all of this tissue along with the rest of the thyroid. The contralateral lobe is then resected in a similar fashion.

Once the gland is resected, one should place the patient in the Trendelenburg position and ask the anesthesiologists to increase the airway pressure in order to ensure hemostasis. Closure is carried out in layers, approximating the sternothyroid and cervical fascia in the midline, followed by reapproximation of the platysma layer. The skin is generally closed with a running absorbable subcuticular suture followed by steri-strips. There is no need for surgical drains in thyroid surgery.

### Central Compartment Neck Dissection

The management of thyroid cancer involves the surgical clearance of all gross disease at the time of surgery, including clinically involved lymph node metastases. As previously discussed, central neck dissections are done routinely for MTC, and therapeutically in cases of well-differentiated thyroid cancer with nodal involvement either identified pre- or intra-operatively. A central neck dissection involves the clearance of all tissue within the nodal basins, between the superficial and deep layers of the deep cervical fascia, superior to the brachiocephalic artery and inferior to the hyoid bone, between the carotid arteries laterally.^6^ A central neck dissection should also include clearance of the prelaryngeal nodes, pretracheal nodes, and all nodal tissue in the lateral tracheoesophageal grooves.^22^ A small subset of patients will have metastases into the superior mediastinum (level VII), and in such cases the surgeon should remove these nodes to the extent possible through the cervical approach. The central compartment dissection should be done in a meticulous fashion in order to avoid injury to the key structures, most notably the parathyroid glands and the RLNs.

### Lateral Neck Dissection

**Figure 1.**
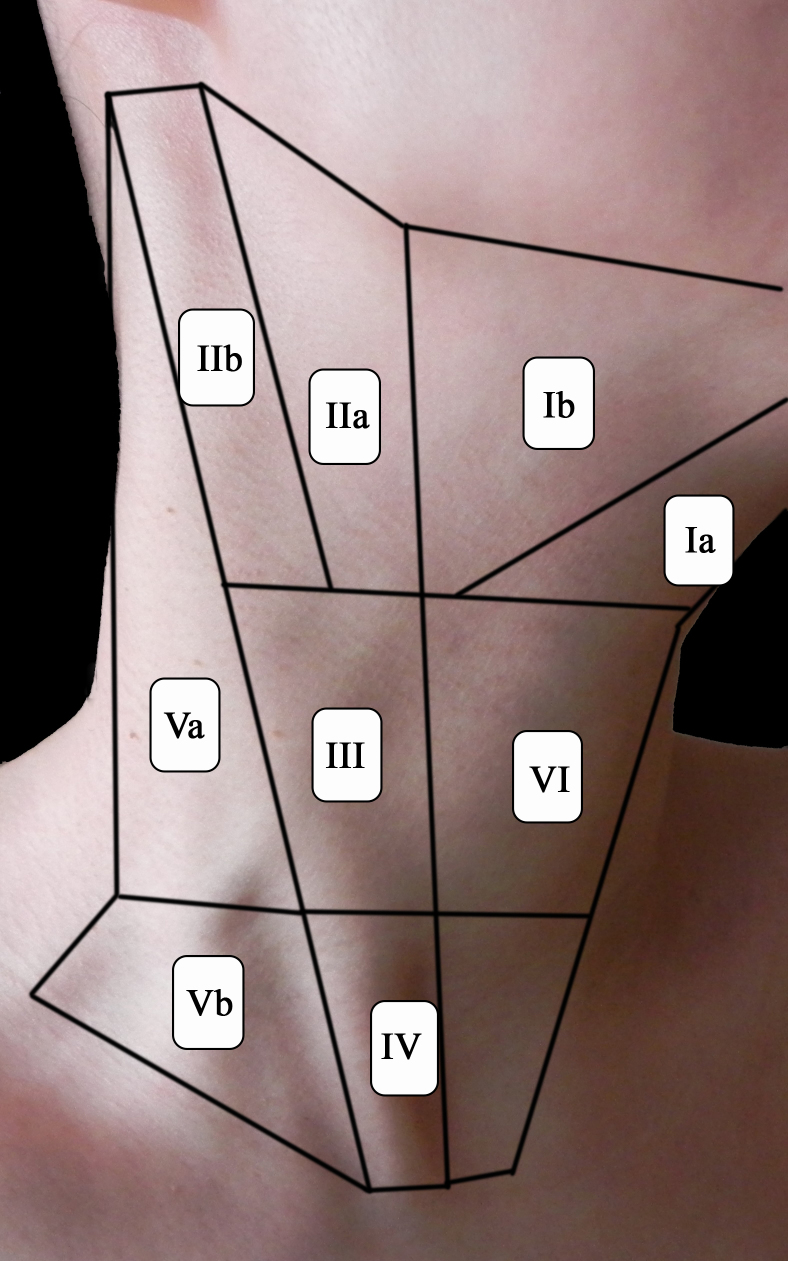
Compartmental Divisions of the Neck.

Prophylactic neck dissection has not been shown to improve survival in patients with differentiated thyroid cancer, and may subject patients to unnecessary operative risk. As such, modified lateral neck dissection is only indicated in the event of clinical involvement of nodal tissue in one or both of the lateral compartments. The lateral neck is traditionally divided into five levels. Level I represents the central superior area, just under the mandible, consisting of the hyoid, stylohyoid, and submandibular gland. It is divided into levels Ia and Ib by the anterior belly of the diagastric muscle. Level I involvement is rare in thyroid cancer; as such this level is not generally included in lateral neck dissections.^23^ Level II is found lateral to level I, corresponding to the base of the skull and angle of the mandible. The tissue planes involve the upper third of the sternocleidomastoid (SCM) muscle, and it is further divided into levels IIa and IIb by the course of the spinal accessory nerve. Levels III and IV follow the SCM muscle inferiorly and include the common carotid arteries laterally. Level IV extends inferiorly to the clavicle. Level V refers to the tissue lateral to the SCM muscle along the trapezius and is further subdivided into Va and Vb at the level of the inferior pole of the cricoid cartilage (Figure 1)

Levels IIa, III, IV, and Vb are typically included in a lateral neck dissection. The thyroidectomy incision is extended laterally (continuing the transverse incision that is placed in an identifiable natural skin crease) with subcutaneous flaps raised further laterally, bringing the SCM muscle into the operative field. Care must be taken to avoid injury to the spinal accessory nerve. The SCM muscle is reflected laterally and superiorly such that adequate exposure of the spinal accessory nerve is achieved. Improved exposure of level IV tissue is attained by division of the omohyoid muscle. The specimen should be removed en bloc in an avascular plane on top of the underlying deep fascia, avoiding injury to the carotid artery, jugular vein, vagus nerve, spinal accessory nerve, and phrenic nerve. The compartment deep to the carotid arteries and internal jugular veins is an area where nodal disease is frequently missed in differentiated thyroid cancer and thus must be fully explored.

Complications of lateral neck dissection include potential nerve injury to the spinal accessory nerve, the phrenic and vagus nerves, as well as the cervical sympathetic chain at the level of the carotid sheath. Risk of injury can be minimized by meticulous dissection in these areas. Numbness of the lateral neck and ear is the most frequently reported complication, which may result from injury to the greater auricular nerve and cervical sensory nerve rootlets. Chyle leaks may also occur in the event of injury to the thoracic duct, posterior to the internal jugular vein on the left, or interruption of lymphatic ducts on the right.^23^

### Management of Parathyroid Glands

Transient (5%) and permanent (1%) hypoparathyroidism is a well-known complication during total thyroidectomy. This complication is due to inadvertent devitalization of all parathyroid glands by either removal or devascularization during the dissection, and the risk for such complication can be increased in advanced-stage cancer operation and central neck dissection. Hypoparathyroidism can manifest with neuromuscular symptoms to life-threatening cardiac complications and, therefore, should be monitored and treated appropriately.^24^–^26^

Each parathyroid gland should be carefully dissected while preserving its blood supply. Normal glands are usually ∼5 mm in size and weigh about 30 to 50 milligrams. The superior glands are embryologically derived from the fourth branchial pouch and lie posterior to the recurrent laryngeal nerve. Inferior glands are derived from the third branchial pouch and lie anterior to the nerve. Ectopic glands may be intra-thyroidal or located in the thymus, carotid sheath, retroesophageal, anterior mediastinum, or pericardium. Some patients may have supernumerary glands. Superior and inferior glands are usually located within 1 cm from the crossing point of the inferior thyroid artery and the recurrent laryngeal nerve.^24^–^26^

Parathyroid autotransplantation should be performed if the gland’s viability is questionable or the gland has been dissected off the vascular pedicle, especially during a challenging dissection for intracapsular or intra-thyroidal glands. In general, superior glands are easier to preserve *in situ* during thyroid cancer surgery, whereas the inferior glands are more often caught up with the tumor or central lymph nodes, making these glands more difficult to find and preserve. Of note, frozen section analysis should be done to confirm the parathyroid tissue while ruling out cancer, lymph node, or residual thyroid tissue prior to autotransplantation. Transplantation can be performed into a strap muscle or the sternocleidomastoid muscle by creating a pocket(s) in the muscle and implanting the cold-saline-preserved gland after mincing it into multiple tiny fragments. The pocket can be closed with a permanent suture or a clip. Implanted gland tissues will induce neovascularization and typically regain function in several weeks.^27^,^28^

## FOLLOW-UP

Postoperative patients can be followed with annual history and physical exams, serum thyroglobulin (Tg), and US imaging. ^131^I imaging can be used in the follow-up of high-risk patients or in patients who demonstrate concern for recurrence or potential new disease.

The use of radioactive iodine (RAI) ablation treatment in the postoperative management of thyroid cancer patients is somewhat controversial. It is used with the aim of eradicating all remnants of normal thyroid tissue as well as any disease, including potentially involved nodal beds. In addition, RAI treatment facilitates postoperative screening with serum Tg. The 2009 ATA Guidelines currently recommend the use of RAI postoperatively in patients with T3, T4, or M1 disease.^3^ Recent literature suggests that the use of RAI in patients with high-risk variants of papillary thyroid cancer (PTC) can prolong survival.^29^

## AREAS OF CONTROVERSY IN SURGICAL MANAGEMENT

### Lobectomy versus Total Thyroidectomy for Papillary Thyroid Microcarcinoma

Papillary thyroid carcinoma is the most common subtype of thyroid cancer, generally associated with an excellent overall prognosis.^30^ A microcarcinoma of the thyroid is defined as a tumor of less than 1 cm, which falls under the classification of T1a by the current American Joint Committee on Cancer. Microcarcinomas account for roughly 40% of all papillary thyroid cancers.^31^ A recent SEER review found a disease-specific survival for PTC microcarcinoma to be 99.3%.^32^ Similarly, patients with these microcarcinomas have low rates of locoregional recurrence and distant metastasis, <6% and 3%, respectively.^31^,^32^

Traditionally, all patients with a preoperative diagnosis of thyroid cancer underwent a total thyroidectomy at the initial time of operation. If the diagnosis was unknown preoperatively, it was common practice to start with a lobectomy and upon positive findings for malignancy proceed with a completion lobectomy at a later date. Given the overall prognosis of small papillary cancers, and their frequent incidental findings on postoperative pathology, the necessity of a total thyroidectomy in these patients has come under question.

Advantages conferred by lobectomy include the avoidance of lifelong thyroid replacement therapy, in addition to lower overall surgical risks of nerve injury and hypoparathyroidism, by avoiding a bilateral operation. However, total thyroidectomy has long been established as the standard of care for all cancers, and, with thyroid tissue present during the patient’s follow-up period, screening for recurrences becomes more difficult. Serum Tg levels and ^131^I scans would not have the same functional significance in this setting.^32^

Recent analysis of the NCDB, the largest available database, over the period from 1985 to 1998, reported the outcomes of approximately 12,520 patients diagnosed with PTC microcarcinoma. At 70 months of follow-up, there was no difference found in either recurrence rate or survival in patients treated by either lobectomy or total thyroidectomy, with respective *P* values of 0.24 and 0.83.^33^

In a large series from the Gustave Roussy Institute, outcomes were compared for unifocal papillary microcarcinoma in patients treated by lobectomy or total thyroidectomy. Both treatment strategies proved to be very effective; patients undergoing lobectomy were observed to have a locoregional recurrence rate of 3.3%. No recurrence was observed in patients treated by total thyroidectomy.^34^

The current consensus provided by the NCCN 2013 and ATA 2009 Guidelines indicate that lobectomy alone is acceptable for papillary microcarcinoma if the following criteria are met: tumors in patients without medical risk factors should be unifocal, confined to the thyroid without extension, demonstrate non-aggressive histology, without lymphovascular invasion, or gross remaining disease following surgery.^3^,^35^

Total thyroidectomy remains the recommended treatment of choice for papillary microcarcinomas with high-risk features, such as nodal involvement, extra-thyroidal extension, multifocality, aggressive histologic variants, lymphovascular invasion, and residual macroscopic disease following surgery.

### Prophylactic Central Neck Dissection

Currently, there are no prospective, randomized trials comparing prophylactic to therapeutic central lymph node dissection. Current practice guidelines from the ATA recommend therapeutic central or lateral lymph node clearance in papillary thyroid cancers for clinically positive nodes. But the guidelines regarding prophylactic neck dissections are less clear. The ATA has said that prophylactic neck dissection “may be performed,” particularly in patients with T3 or T4 tumors, though dissection “may be reasonably avoided” for patients with T1 or T2 disease.^3^ Furthermore, the role of preoperative genetic mutational status (e.g. BRAF, RET/PTC, etc.) are also controversial at this point.

Proponents of prophylactic central neck dissection cite the frequent involvement of cervical lymph nodes in thyroid cancer, in addition to the fact that preoperative imaging and the operating surgeon are frequently unable accurately to distinguish positive lymph nodes in the central compartment. In a recent study by Noguchi et al., where patients routinely underwent systematic node dissection, 80% of pathologically positive nodes in the study were found to be misjudged by the operating surgeon as being clinically negative.^36^,^37^

The removal and adequate identification of involved lymph nodes improves the accuracy of staging patients with thyroid cancer; however, this may not routinely affect management or overall survival. Lymphadenectomy is relatively safe to perform at the time of the initial operation, but re-operation, especially in the central neck compartment, is associated with an increased risk to the RLN and parathyroid glands. Additionally, it is unclear whether RAI is effective in eliminating residual disease in the central or lateral lymph node basins.

Frequently cited reasons to avoid routine lymphadenectomy include exposing patients to an unnecessary increased risk of nerve injury and hypoparathyroidism. Additionally, lymph node involvement does not appear to impact recurrence. In a series of 300 patients that did not undergo dissection, Noguchi et al. reported no recurrences.^36^,^37^ High rates of disease-free survival and overall survival are frequently observed, irrespective of dissection practices. As such, central and lateral neck dissections should generally be performed only in patients with clinically positive nodes.

## SUMMARY

With the increasing incidence of thyroid cancer, there has been a similar increase in development and utilization of multidisciplinary tools to assist in clinical management, such as the growth of genetic panels, incorporation of tumor biology into screening, improved diagnostic imaging, and the standardized TIRADS classification system. New controversies have emerged in surgical practice, such as the role of prophylactic neck dissection in well-differentiated thyroid cancers. While surgery remains the center of treatment for most patients with thyroid cancer, an increasing knowledge base and experience in the multidisciplinary management of thyroid cancer will be required.

## Abbreviations:

AACE: American Association of Clinical Endocrinologists
ATA: American Thyroid Association
ATC: anaplastic thyroid cancer
DM: diabetes mellitus
FC: follicular thyroid cancer
FNA: fine-needle aspiration
MTC: medullary thyroid cancer
NCCN: National Comprehensive Cancer Network
PTC: papillary thyroid cancer
RLN: recurrent laryngeal nerve
SCM: sternocleidomastoid
Tg: thyroglobulin
US: ultrasound.
